# Squamous Cell Carcinoma in the Post Tuberculosis Lung after 30 Years of Treatment Completion

**DOI:** 10.1155/2020/8570212

**Published:** 2020-01-30

**Authors:** Arulprashanth Arulanantham, Umesh Jayarajah, Rohitha Dharmasiri, Rasarathinam Jeyanthakumar, Kamila Niroshan Siriwardena, Sujeewa Ilangamge

**Affiliations:** ^1^National Hospital of Sri Lanka, Colombo, Sri Lanka; ^2^National Hospital for Respiratory Diseases, Welisara, Sri Lanka

## Abstract

Pulmonary tuberculosis (TB) and lung cancer are becoming increasingly prevalent especially in developing countries. The occurrence of lung cancer after 30 years of completed pulmonary TB treatment is rare. We report a rare occurrence of a squamous cell carcinoma (SCC) in the post TB lung after 30 years of completed pulmonary tuberculosis treatment. A 60-year-old male, an apparently healthy nonsmoker, presented with a community-acquired Klebsiella pneumonia. Imaging revealed a destroyed left lung with cavities with air-fluid levels. An enhancing lesion was noted at the left upper lobe, and a guided biopsy revealed a SCC. He was fit for surgery and underwent an open left pneumonectomy. The left lung was destroyed and cavitatory due to the previous tuberculosis. A peripherally located tumor was noted in the left upper lobe. Histology revealed a moderately differentiated keratinizing type SCC (pT4N0Mx). The negative cultures and histology excluded an active pulmonary tuberculosis. The postsurgical lung function at 1 month showed satisfactory improvement with good functional capacity. He was then referred to the oncologist for adjuvant therapy. The occurrence of post-TB lung cancer after 30 years in an otherwise healthy male without active TB suggests an increased long-term risk of cancer even in the absence of other robust risk factors. Therefore, the chronic inflammatory process in the diseased lung is probably the cause for lung cancer in the absence of active TB. Thus, we suggest long-term surveillance after completed pulmonary TB treatment even in otherwise healthy asymptomatic individuals.

## 1. Introduction

Lung cancer and pulmonary tuberculosis (TB) are becoming increasingly prevalent especially in the developing countries leading to a significant burden to the health services [1, 2]. Furthermore, lung cancer and pulmonary tuberculosis are known to coexist especially in regions having a higher prevalence of pulmonary TB [3–5]. Both diseases share common risk factors and clinical features. Pulmonary TB predicts an increased risk for lung cancer probably due to the chronic inflammatory process occurring in the lungs [3–5]. Fortunately, pulmonary TB does not appear to alter the prognosis and the clinical course of lung cancer if treated properly [3–5].

Usually, lung cancers are detected in the first five years after the diagnosis of pulmonary TB and are seen in smokers [1]. The occurrence of lung cancer after 30 years of completed pulmonary TB treatment is rare. We report a rare occurrence of a squamous cell carcinoma in the post-TB lung after 30 years of completed pulmonary tuberculosis treatment in an otherwise healthy nonsmoker.

## 2. Case Presentation

A 60-year-old male, with a history of completed open pulmonary TB treatment 30 years ago, presented with fever with productive cough and left-sided pleuritic chest pain over 2 weeks. He is a nonsmoker, and his past medical history was otherwise unremarkable and was completely asymptomatic up to this presentation. He was a manual laborer and had a good exercise tolerance. He was thin built but did not have any features of nutritional deficiency. His respiratory rate was 16/min. He had reduced left-sided chest expansion and prominent left apical flattening. His trachea was shifted to the left side and auscultation revealed reduced air entry with a dull percussion noted on the left lower zone. His systemic examination was unremarkable.

**Figure 1:**
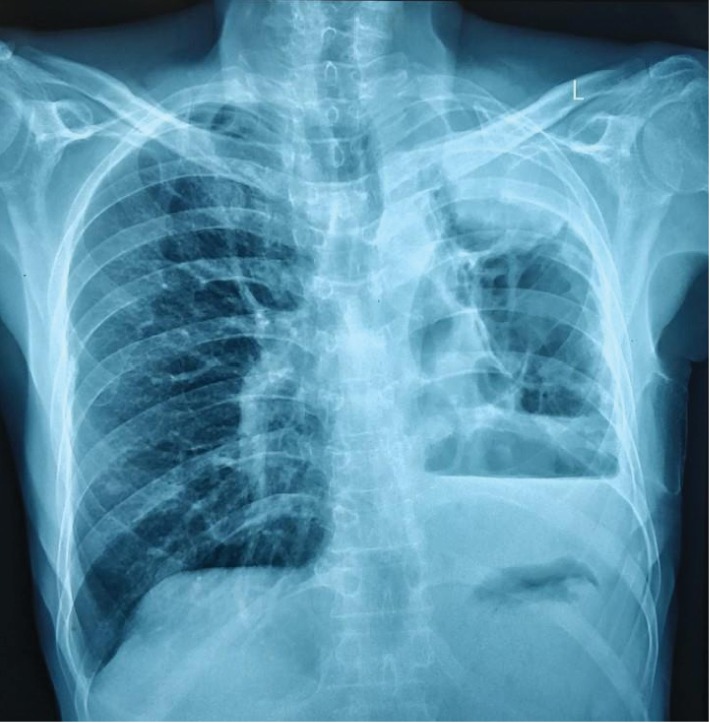
Chest X-ray showing loss of lung volume on the left side with a mediastinal shift. There were large cavities in the left upper and middle zones with an opacity at left upper zone and left-sided moderate effusion.

His chest radiograph (Figure 1) showed a loss of lung volume on the left side with a mediastinal shift. There were large cavities in the left upper and middle zones with an opacity at the left upper zone and left-sided moderate effusion. His sputum culture was positive for Klebsiella and was treated as Klebsiella pneumonia. Moreover, the sputum was negative for acid fast bacilli and culture was negative for TB.

**Figure 2:**
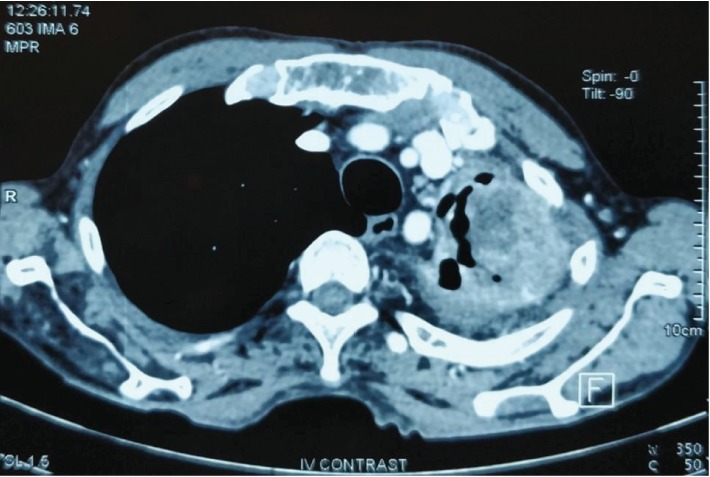
CT scan (mediastinal window) showing an enhancing lesion at the apicoposterior segment of the left upper lobe measuring 6.8 × 4.8 × 3.8 cm.

**Figure 3:**
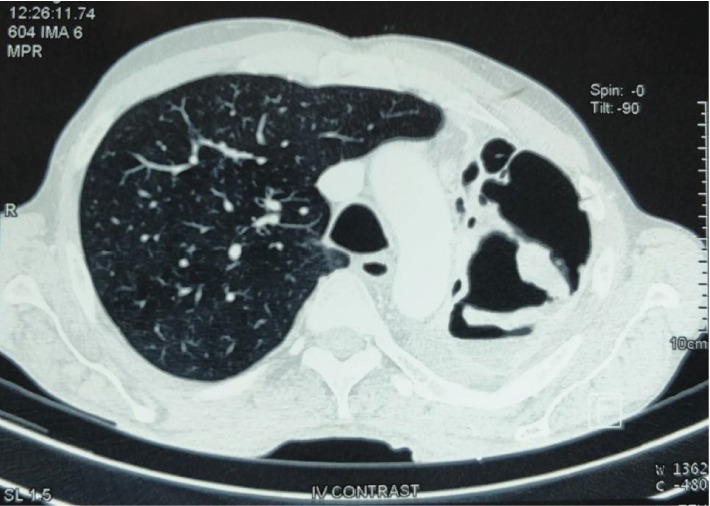
CT scan (lung window) showing a completely destroyed left lung with multiple air- and fluid-filled cavities and fibrotic bands.

The contrast-enhanced computed tomography (CT) scan revealed a completely destroyed left lung. There were multiple air- and fluid-filled cavities and fibrotic bands. An enhancing lesion was noted at the apicoposterior segment of the left upper lobe measuring 6.8 × 4.8 × 3.8 cm with marginally enlarged lymph nodes in the aortopulmonary window. The right lung fields were expanded with a mediastinal shift (Figures 2 and 3).

Fibrooptic bronchoscopy excluded a major bronchial involvement of the lesion. Ultrasound guided biopsy was suggestive of a malignancy with a squamous cell variant.

He had satisfactory cardiac and lung functions. Considering his fitness for surgery and completely destroyed lung with a left upper lobe malignancy, a left pneumonectomy was decided.

**Figure 4:**
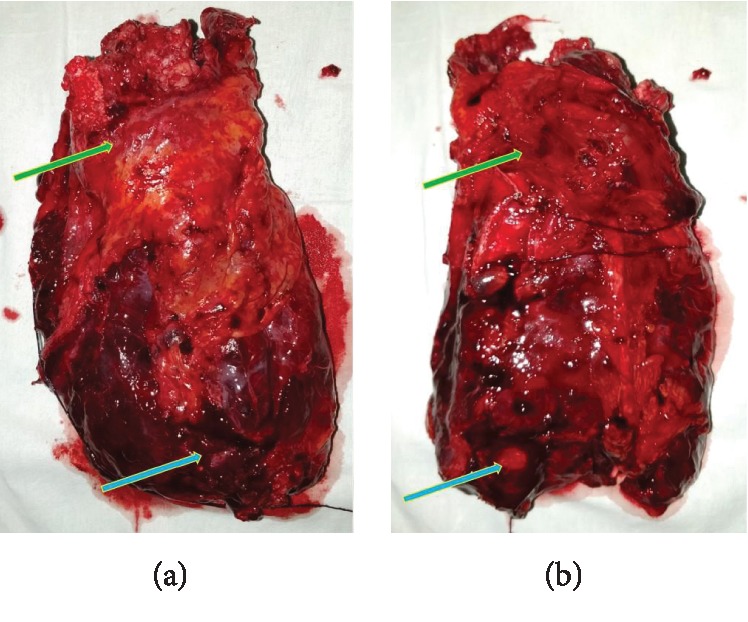
(a) Anterior view and (b) posterior view of the left lung. A destroyed, small, cavitatory left lung with the tumor in the upper lobe (green arrow) and empyema around the lower lobe (blue arrow).

He underwent a left posterolateral thoracotomy, and the operative findings mirrored the CT findings. The peripherally located tumor was confined to the left upper lobe. The left lung was destroyed, cavitatory, and small due to the previous tuberculosis. An empyema with a large cavity around the lower lobe was noted. The pus culture was negative for TB. Several mediastinal lymph nodes on the aortopulmonary window were enlarged and were resected out. Although the left lung had significant adhesions, the parietal pleura was free of tumor. The hilar vessels and the left main bronchus were divided separately using endostaplers, and the left lung was removed en bloc (Figure 4). He was given postoperative intensive care, and his recovery was unremarkable. He was discharged on postoperative day 5 on pulmonary rehabilitation.

Histology revealed a moderately differentiated keratinizing-type squamous cell carcinoma with a maximum diameter of 7.5 cm (T4) and visceral pleural invasion (PL1). Bronchial resection margin was free of tumor with a 10 mm clearance (pT4N0Mx). All 16 lymph nodes were free of tumor. There were no features to suggest active TB. The post surgical lung function at 1 month showed satisfactory improvement with good functional capacity. He was then referred to the oncologist for adjuvant therapy.

## 3. Discussion

The occurrence of pulmonary TB and lung cancer in the same patient both simultaneously and sequentially has been described in literature [3–5]. The association between pulmonary TB and lung cancer is important as both diseases are becoming increasingly prevalent in developing countries [3–5]. We describe the rare occurrence of a keratinizing squamous cell carcinoma in the post-TB lung after 30 years of completed pulmonary TB treatment in an otherwise well nonsmoker. He presented with a community-acquired Klebsiella pneumonia in a post-TB lung with multiple cavitatory lesions, an upper lobe nodule, and pleural effusion. The diseased left lung was almost completely destroyed. A prominent mediastinal shift and compensatory expansion of the right lung were noted.

Pulmonary cavities and nodules are common radiological findings of various etiologies. Pulmonary TB is the commonest cause, and other common causes include lung cancer and pulmonary aspergillosis. The CT findings are helpful in the differentiation; however, it is difficult to interpret in a diseased lung. Malignancies usually include a thicker wall (at least over 4 mm in the thinnest part), irregular inner and outer linings, associated soft tissue mass with surrounding infiltration, and enlarged regional lymph nodes [6]. Conversely, tuberculous cavitatory lesions are typically located in the upper lobes or superior segments of the lower lobes. The thickness may vary, and there may be air-fluid levels [6], whereas in aspergillosis, there may be thick-walled cavitatory lesions in the upper lobes which may be solitary or multiple. The cavity walls may be thick or thin, with or without air-fluid levels [6]. In our patient, the diagnosis of lung cancer was difficult through imaging due to the preexisting diseased lung complicated by pneumonia. Therefore, histological confirmation was needed for the diagnosis.

Lung cancer may coexist with pulmonary TB or may develop subsequently. In our patient, there was subsequent development of lung cancer after 30 years without any evidence of active pulmonary TB. Several theories have been proposed to explain the pathogenesis of post-TB lung cancer. Post-TB scars may deform blood and lymphatic vessels leading to lymphostasis causing deposit of carcinogen and promoting malignant processes [3–5]. Furthermore, chronic inflammation associated with recurrent infections may lead to carcinogenesis due to production of reactive species by activated neutrophils and macrophages causing genetic alterations and malignant transformations [3–5]. Such mutations in the fragile histidine triad gene have been suggested in the pathogenesis of lung cancer following pulmonary TB [3–5]. Furthermore, increased cellular proliferation during the repair process in chronic inflammation may trigger metaplasia and subsequent neoplastic changes [3–5]. Other postulated mechanisms for coexistence of pulmonary TB and lung cancer include reactivation of TB by carcinoma due to immune suppressions and secondary infection of the carcinoma by TB [4].

Liang et al. in their systematic review investigated several confounding factors related to pulmonary TB and lung cancer. They found that the risk association of pulmonary TB and lung cancer is seen even in nonsmokers. Furthermore, chronic inflammation and metaplasia followed by neoplastic changes have been proposed as the causative mechanisms [7]. The risk of carcinoma was highest in the first five years following the diagnosis of pulmonary TB; however, the risk prevailed even up to 20 years following diagnosis of pulmonary TB [1]. Nevertheless, the development of lung cancer after more than 30 years of completed TB treatment is a rare occurrence [1].

Several studies have suggested that lung cancer following pulmonary TB is not of bronchial in origin and rather arise from the scar tissue [8, 9]. Furthermore, upper lobes are commonly affected compared to other regions [8, 9]. Literature review revealed conflicting evidence on the histological subtypes of lung cancer related to TB. A large retrospective cohort study by Cicenas and Vencevicius found that the majority of concurrent lung cancer were squamous cell carcinoma [3]. Furthermore, a study by Varol et al. also showed that squamous cell carcinoma was the predominant type [10]. However, both studies looked at lung cancer coexisting with pulmonary TB. A systematic review on preexisting TB and lung cancer risk showed that only adenocarcinoma was associated with TB [1]. Furthermore, studies have shown that cancer occurring in a scarred lung were usually adenocarcinoma [8, 9]. Based on the above literature, it seems that squamous cell carcinoma is common in concurrent cancers and adenocarcinoma in subsequent cancers.

The association of lung cancer in the post-TB lung is well established. This is probably due to the shared risk factors such as smoking, chronic obstructive pulmonary disease, and immunosuppression. We reported an otherwise healthy nonsmoker who developed lung cancer in a post-TB lung after 30 years of completed pulmonary TB treatment without any evidence of active disease. Therefore, an increased long-term risk of cancer is seen in such patients even in the absence of other robust risk factors. The chronic inflammatory process in the diseased lung is probably the underlying mechanism even in the absence of active TB. Therefore, we suggest long-term surveillance after completed pulmonary TB treatment even in otherwise healthy asymptomatic individuals.
